# Buzhongyiqi Decoction Protects Against Loperamide-Induced Constipation by Regulating the Arachidonic Acid Pathway in Rats

**DOI:** 10.3389/fphar.2020.00423

**Published:** 2020-04-03

**Authors:** Wan-Jun Ju, Ze-kuo Zhao, Shao-Li Chen, Dan-dan Zhou, Wen-Ning Yang, Xiao-Ping Wen, Guang-Li Du

**Affiliations:** ^1^Department of Formulaology, School of Basic Medical Sciences, Shanghai University of Traditional Chinese Medicine, Shanghai, China; ^2^Department of Endocrinology, Shanghai Pudong New Area Hospital of Traditional Chinese Medicine, Shanghai, China; ^3^R & D Department, GenChim Testing Co., Ltd, Shanghai, China; ^4^School of Chinese Materia Medica, Beijing University of Chinese Medicine, Beijing, China

**Keywords:** constipation, Buzhongyiqi decoction, Metabolomics, Inflammation, rat

## Abstract

Constipation is a common gastrointestinal disorder without effective treatment approach. Buzhongyiqi decoction (BZYQD) is a classical formula that has been commonly used for gastrointestinal disorders for nearly 1,000 years. In this study, we aimed to investigate the protective effect of BZYQD against loperamide-induced constipation and its potential mechanism. Rats with loperamide-induced constipation were orally administered BZYQD. BZYQD treatment obviously increased the small intestinal transit rate and alleviated colon tissue pathological damage. Subsequently, serum metabolomics study was performed to identify the metabolites affected by BZYQD. Metabolomics identified that the levels of 17 serum metabolites, including prostaglandin E_2_ (PGE_2_), arachidonic acid (AA), and inositol, were significantly changed in BZYQD-treated group compared with those in the loperamide-induced group. Pathway analysis revealed that those metabolites were mainly associated with arachidonic acid metabolism, biosynthesis of unsaturated fatty acids, ascorbate and aldarate metabolism, inositol phosphate metabolism. Additionally, BZYQD treatment down-regulated the cyclooxygenase-2 expression and decrease production of the proinflammatory mediator PGE_2_. Further study revealed that BZYQD administration decreased serum levels of the inflammatory factors IL-1β and TNF-α, inhibited phosphorylation of the nuclear transcription factor NF-κB, and down-regulated expression of the inflammatory factors IL-1β and IL-6 in the constipated rat colon. Moreover, BZYQD treatment also increased serum levels of inositol, motilin and gastrin, and promoted gastrointestinal motility. In conclusion, the present study suggested that BZYQD exerted a protective effect against loperamide-induced constipation, which may be associated with its role in regulation of multiple metabolic pathways.

## Introduction

Constipation is a clinically common gastrointestinal dysfunction with a prevalence of 5–20% worldwide (Suares and Ford, 2011). According to the Rome IV criteria, a typical symptom of chronic constipation is difficult, infrequent, or inadequate bowel movement (Mearin et al., 2016). Individuals with bowel movement every 3–4 days are at a higher risk of colon cancer, hemorrhoids, and other gastrointestinal diseases (Wu et al., 2010). Laxatives are widely prescribed as the main means to assist patients in passing stools (Li et al., 2019). However, these treatments have severe side effects. Therefore, development of more efficient and safe therapeutic/preventive agents and methods is still needed.

Traditional Chinese medicine (TCM) formulas have been widely used for the prevention and treatment of digestive diseases for thousands of years. Buzhongyiqi decoction (BZYQD) is a well-known TCM formula first described in Pi Wei Lun, a treatise on digestive system diseases written by the famous Chinese physician Li Gao (1180–1251 A.D. of the Chinese Yuan Dynasty) (He et al., 2017). BZYQD is comprised of eight herbs (**Table 1**) (Ji, 2006) and has been identified as an effective drug for improving the digestive system function, quality of life, and nutritional status in elderly patients with chronic obstructive pulmonary disease (Gou et al., 2016). Moreover, BZYQD is also a representative prescription that is increasingly applied to treat gastrointestinal dysfunction, such as constipation, in China (Wang et al., 1991; Sun Feng-Wei, 2016; Zhang, 2016; Dan, 2018). However, its mechanism of action has not yet been fully investigated.

**Table 1:** Herbal constituents of BZYQD.

Pharmaceutical name	Chinese name	Part used	Amount (g)
*Astragalus mongholicus* Bunge	Huangqi	Root	18
*Glycyrrhiza uralensis* Fisch. ex DC	Gancao	Root and rhizome	9
*Codonopsis pilosula* (Franch.) Nannf.	Dangshen	Root	9
*Angelica sinensis* (Oliv.) Diels	Danggui	Root	3
*Citrus ×* aurantium L.	Chenpi	Pericarp	6
*Actaea heracleifolia* (Kom.) J. Compton	Shenma	Rhizome	6
*Bupleurum chinense* DC.	Chaihu	Root	6
*Atractylodes macrocephala* Koidz.	Baizhu	Rhizome	9

Metabolomics represents a powerful discipline concerned with the comprehensive analysis of small molecules to discover biomarkers in biological systems (Schrimpe-Rutledge et al., 2016; Zhang et al., 2019). This method provides holistic insights into changes in the metabolic pathways during disease or drug treatment (Wu et al., 2017; Zheng et al., 2019). Therefore, metabolomics study is in perfect accordance with the holistic approach of TCM, and can provide clues to mechanism of action of TCM (Wu et al., 2017; Zheng et al., 2019). In the current study, the effect of BZYQD on loperamide-induced constipation in a rat model was investigated. Additionally, the underlying molecular mechanism of BZYQD in constipation was explored by serum metabolomics. Our results provide novel insights into the potential role of the herbal medicine BZYQD in treating gastrointestinal dysfunction diseases.

## Materials and Methods

### Chemical Compounds and Reagents

Chromatography-grade acetonitrile was purchased from Merck (Darmstadt, Germany). Water was purified using a Milli-Q water system (Millipore, Bedford, MA, USA). Chromatography-grade acetic acid, formic acid, and methanol were provided by Tedia Company (Fairfield, OH, USA). All the crude drugs including Radix Astragali (Huangqi in China, HQ), Radix Glycyrrhizae (Gancao in China, GC), Rhizoma Atractylodis Macrocephalae (Baizhu in China, BZ), Radix Codonopsis (Dangshen in China, DS), Radix Angelicae Sinensis (Danggui in China, DG), Pericarpium Citri Reticulatae (Chengpi in China, CP), Radix Bupleuri (Caihu in China, CH), and Rhizoma Cimicifugae (Shengma in China, SM) were purchased from Shanghai Kangqiao Chinese Medicine Tablet Co., Ltd (Shanghai, China) and authenticated by Gen Chim Testing Co., Ltd (Shanghai, China). Mosapride was purchased from Sigma-Aldrich (St. Louis, MO, USA).

### Preparation and Analysis of BZYQD

**Figure 1:**
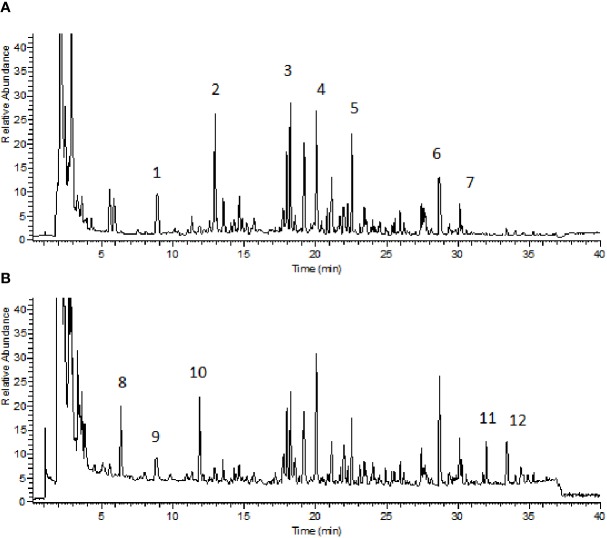
Total ion chromatogram of BZYQD extract in negative-ion mode **(A)** and positive-ion mode **(B)**. 1: Piscidic acid; 2: Ferulic acid; 3: Liquiritin; 4: Hesperidin; 5: 2-feruloylpiscidic acid; 6: Glycyrrhizic Acid; 7: Saikosaponin D; 8: Synephrine; 9: Codonopyrrolidium B; 10: Tryptophan; 11: Nobiletin; 12: 3, 5, 6, 7, 8, 3', 4'-heptamethoxyflavone.

BZYQD was prepared according to our previously reported method. A mixture of HQ (18 g), DS (9 g), BZ (9 g), DG (3 g), GC (9 g), CP (6 g), SM (6 g), and CF (6 g) was added to 600 ml water and extracted at 100°C for 1 h. The extracted solution was filtered and spray-dried to obtain dry extract powder. The final ratio of BZYQD extract powder to raw herb was 37.80%. The total ion chromatogram of BZYQD extract that measured by LC-MS is shown as **Figure 1**.

### Animals and Experimental Design

**Figure 2:**
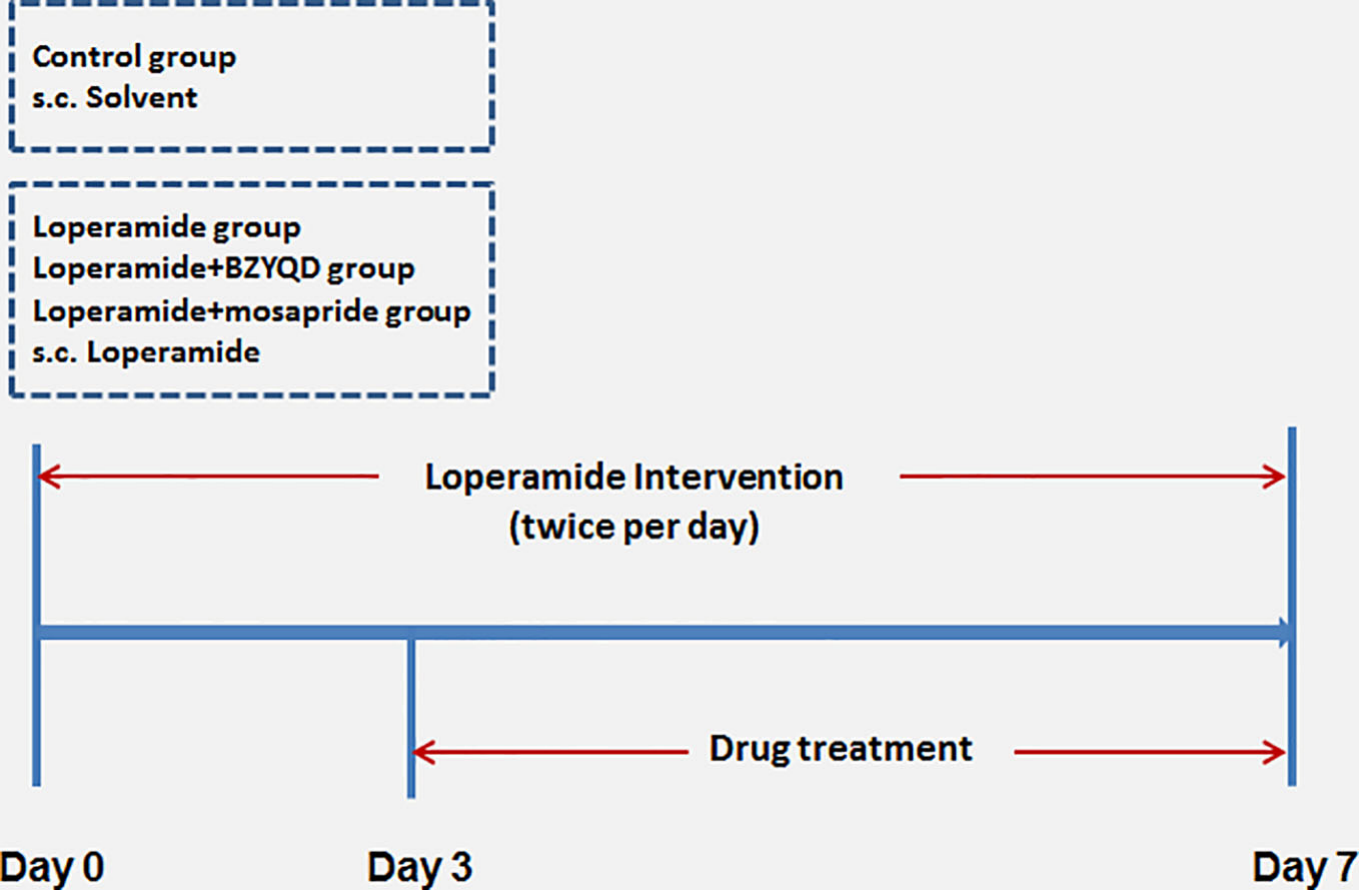
Scheme of experiment. Buzhongyiqi decoction (BZYQD) treatment commenced on day 3, s.c: subcutaneous injection.

Male 8-week-old Sprague–Dawley (SD) rats were provided by the Laboratory Animal Center of Shanghai University of Traditional Chinese Medicine. The animals were housed and acclimatized for one week prior to the experiment in a room at 20–22°C and 60–70% humidity under a 12 h-light and 12 h-dark cycle. All animal experiments were carried out in full compliance with the Guidance of Humane Care and Use of Laboratory and approved by the Committee on the Use of Live Animals for Teaching and Research in the Shanghai University of Traditional Chinese Medicine. Rats were randomly divided into four groups: control (normal control), loperamide, loperamide+BZYQD (1.73 g/kg of BZYQD extract powder), and loperamide+mosapride (positive group, 1.6 mg/kg), with 8 rats per group. Rats of the loperamide, loperamide+BZYQD, and loperamide+mosapride groups were treated with loperamide (4 mg/kg, b. w.), by subcutaneous injection twice per day at 09:00 and at 18:00 h for 1 week, and all the corresponding drugs was orally administered to the rats once daily from the third day. Rats in the control group and loperamide group orally received the normal saline. The scheme of the animal experiment is summarized in **Figure 2**. A 100% survival rate was recorded at the end point of the experiment.

### Measurement of Gastro-Intestinal Propulsion (GIP)

All the groups were exposed to fasting conditions and drank only water on the seventh day. GIP was measured through prepared Chinese ink. Thirty minutes later, all animals were fed with the prepared Chinese ink (10% charcoal in 5% gum Arabic) and were anesthetized. The small intestine was immediately dissected and put on a clean surface. The travelling distance of the charcoal meal from the pylorus was measured. The gastrointestinal propulsion was calculated based on the following formula: GIP = (Distance travelled by charcoal (cm)/total intestinal length (cm) ×100%. Then, the colon tissues were collected for further studies.

### Hematoxylin-Eosin Staining

Small intestine and colon tissues were fixed in 10% formaldehyde, dehydrated, embedded in paraffin, cut into 5-μm-thick sections, and stained with hematoxylin and eosin. Pathological changes were observed using the OlympusBX70 research microscope.

### Serum Metabolomics Study

#### Sample Preparation

An aliquot of 100 μl acetonitrile containing the internal standards (IS, 10 μg/ml 2-chloro-L-phenylalanine in positive and negative modes) was added to 20 μl of serum samples. After vortex-mixing for 3 min, the mixture was centrifuged for 10 min at 16,000 rpm at 4°C, and 5 μl of the supernatant was injected for UPLC-MS/MS analysis. An equal aliquot from each serum sample was combined and mixed to prepare the quality control (QC) samples. The QC samples were processed by the same method as the other serum samples and analyzed randomly through the analytical batch to ensure the stability of the analytical process.

#### Analysis Conditions

Sample analysis was performed using a UPLC-LTQ-Orbitrap system (Thermo Fisher Scientific, San Jose, CA, USA) and the samples were separated using a Waters ACQUITY UPLC BEH C18 column (2.1×100 mm, 1.7 μm) (Waters, Co., Milford, MA, USA). The mobile phases contained water with 0.1% formic acid (A) and acetonitrile with 0.1% formic acid (B). The elution steps were as follows: 10% B from 0.1–2 min, 10–40% B from 2.1–7 min, 40–80% B from 7.1–11 min, 80–90% B from 11.1–15 min, and 90% B for 0.5 min. At 15.5 min, B was adjusted to 10% and the column equilibrated for 4.5 min. The mass spectrometer parameters were as follows: ion spray voltage, 3.8 kV(+) and 3.2 kV(−); capillary and heater temperature, both 350°C; sheath and auxiliary gas flow rate, 45 and 15 psi, respectively; and S-Lens RF level, 60% (Li et al., 2017; Wu et al., 2017).

#### Data Processing

Data preprocessing, identification of potential biomarkers, and pathway analysis were performed as reported previously (Huang et al., 2013; Li et al., 2017). In brief, the raw data of UPLC-LTQ-Orbitrap were imported to SIEVE to perform peak extraction and matching. Retention time, m/z value, and corresponding intensities were recorded. These data were preprocessed as per the rules below: (1) the percentage of valid data in each group must be higher than 80%; (2) the relative standard deviation (RSD) in QC samples must be lower than 30%; (3) the intensities of each variable should be normalized by the intensity of the IS. After preprocessing, data were imported to SIMCA-P version 14.0 (Umetrics, Sweden) to perform principal components analysis (PCA) and orthogonal partial least squares discriminant analysis (OPLS-DA). The variable importance in projection (VIP) value was calculated in the OPLS-DA model and the Kruskal-Wallis test was conducted. Variables with VIP values more than 1 and P values less than 0.05 were considered potential biomarkers. These potential biomarkers were identified by searching databases such as HMDB (http://www.hmdb.ca/), KEGG (https://www.kegg.jp/), ChemSpider (http://www.chemspider.com/), and mzCloud (https://www.mzcloud.org/), and were finally validated by standard substances to confirm their identity.

### Immunohistochemistry Analysis

Dewaxed, hydrated colon tissue sections were pretreated with antigen retrieval fluid (pH 6.0), incubated with 3% H_2_O_2_ deionized water for 15 min to block endogenous peroxidase, and rinsed with PBS. IL-6 (1:400) and TNF-α (1:400) were added dropwise and samples incubated overnight at 4°C. The sections were immersed in PBS, and an IgG antibody and Fab fragment-HRP multimer were added dropwise. The mixture was incubated at 37°C for 30 min, and the samples were subsequently washed 5 times with PBS for 3 min each time. Samples were rinsed with distilled water, counter stained with hematoxylin, dehydrated with gradient alcohol, cleared with xylene, and sealed with resin. Positive immunostaining in five random visual fields of the slides was evaluated using an Olympus DP72 optical microscope at a magnification of ×200.

### Real-Time Polymerase Chain Reaction (PCR) Analysis

Total RNA was extracted from colon tissues using Trizol, and cDNA was subsequently synthesized using the Super Script cDNA synthesis kit. Real-time polymerase chain reaction (real-time PCR) was performed using the ABI-StepOnePlus sequence detection system (Applied Biosystems, CA, USA) using the Fast SYBR Green mix kit. The primers used are presented in **Table 2**. The relative target mRNA expression levels were calculated using the 2^-ΔΔCt^ method. The expression level of glyceraldehyde 3-phosphate dehydrogenase (*GAPDH*) mRNA was used as the endogenous reference control.

**Table 2:** Primers of real-time PCR assay used in this study.

GeneName	Specise	Primer sequence (5′-3′)
*Gapdh*	Rat	Forward primer AGGTCGGTGTGAACGGATTTTG
		Reverse primer GGGGTCGTTGATGGCAACA
*NF-κB*	Rat	Forward primer GACGACACCTCTACACATAGCA
		Reverse primer CCTCATCTTCTCCAGCCTTCTC
*IL-6*	Rat	Forward primer CCGGAGAGGAGACTTCACAG
		Reverse primer CCATAGTGCAGGAGCGTACAGT
*IL-1β*	Rat	Forward primer TGACCCATGTGAGCTGAAAG
		Reverse primer GGGATTTTGTCGTTGCTTGT
*TNF-α*	Rat	Forward primer TGATCCGAGATGTGGAACTG
		Reverse primer CGAGCAGGAATGAGAAGAGG
*COX-2*	Rat	Forward primer TCTCCAACCTCTCCTACTAC
		Reverse primer GCACGTAGTCTTCGATCACT

### Western Blot Analysis

The colon samples were homogenized in a radioimmunoprecipitation assay buffer. The lysate proteins were separated using sodium dodecyl sulfate-polyacrylamide gel electrophoresis (SDS-PAGE) and were electro-blotted onto nitrocellulose membranes. The membranes were blocked for 1 h, and then incubated overnight with primary antibodies against p-NF-κB and NF-κB (Santa Cruz Biotechnologies, Inc., Santa Cruz, CA, USA) at 4°C. The membranes were subsequently washed with Tris-buffered saline/0.1% (v/v) Tween-20 and incubated for 1 h with secondary antibodies. After washing the membranes, the protein bands were detected using the FluorChem E image detection system (ProteinSimple, San Jose, CA, USA). β-actin was used as a loading control.

### Enzyme Linked Immunosorbent Assay (ELISA)

The protein levels of IL-1β and IL-6 in rat serum were measured using an ELISA kit (AMEKO Institute of Biotechnology, Shanghai, China) according to the manufacturer's instructions.

### Statistical Analysis

Data are expressed as the mean ± standard deviation (SD). The statistical differences among study groups were determined by one-way analysis of variance (ANOVA) followed by the least significant difference (LSD). For all comparisons, *P* < 0.05 was considered a statistically significant difference. Correlation coefficient (r) was determined using the Pearson's correlation.

## Results

### Effect of BZYQD on Constipation in Loperamide Induced Rats

**Figure 3:**
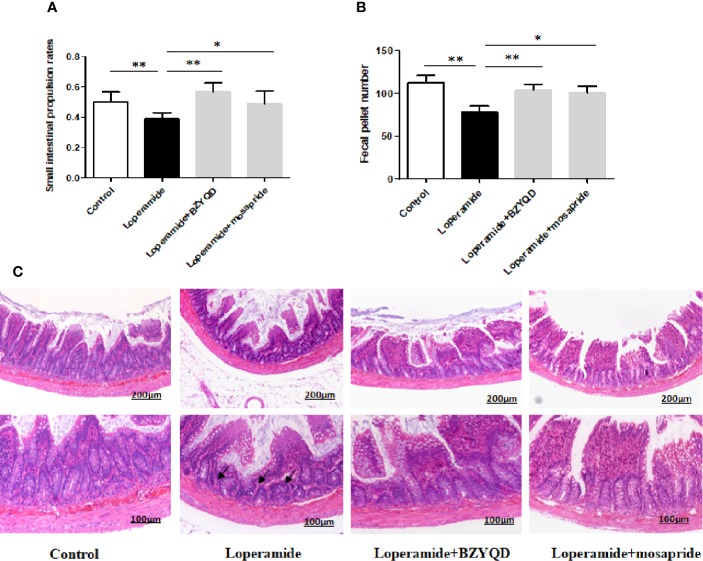
Effect of BZYQD intervention on loperamide-induced constipation in rats. **(A)** Small intestinal transit rates of rats of Control group, Loperamide, Loperamide + BZYQD, and Loperamide + Mosapride groups. **(B)** Number of stools of rats of Control group, Loperamide, Loperamide + BZYQD, and Loperamide + Mosapride groups. **(C)** Hematoxylin and eosin (H&E)-staining for light microscopy analysis of colon sections in rats of the control group, Loperamide group, Loperamide + BZYQD group, Loperamide + Mosapride groups morphology. Arrow marks pointed to that epithelial surface of the rat colon was damaged, mucosa was thinner, gland was reduced, goblet cells were reduced, and the inflammatory cells infiltrated in the lamina propria in the loperamide group. n = 8; Original magnification ×100 or ×200; data are represented as mean ± SD. ^#^*P* < 0.05 versus control group; *P < 0.05versus loperamide group, **P < 0.01 versus Loperamide group.

The body weight did not differ significantly between loperamide-induced group and normal control group (**Supplementary Figure 1**). The small intestinal transit rate and number of stools were significantly decreased in loperamide induced group than those in normal control group (**Figures 3A, B**). Treatment with BZYQD markedly increased the small intestinal transit rates and number of stools as compared to those in the loperamide group (**Figures 3A, B**). As compared with normal control group, the epithelial surface of the rat colon was damaged, mucosa was thinner, gland was reduced, goblet cells were reduced, and the inflammatory cells infiltrated in the lamina propria in the loperamide group; these histological damages were significantly ameliorated by BZYQD treatment (**Figure 3C**).

### Effect of BZYQD on the Metabolism of Rats with Loperamide-Induced Constipation

**Figure 4:**
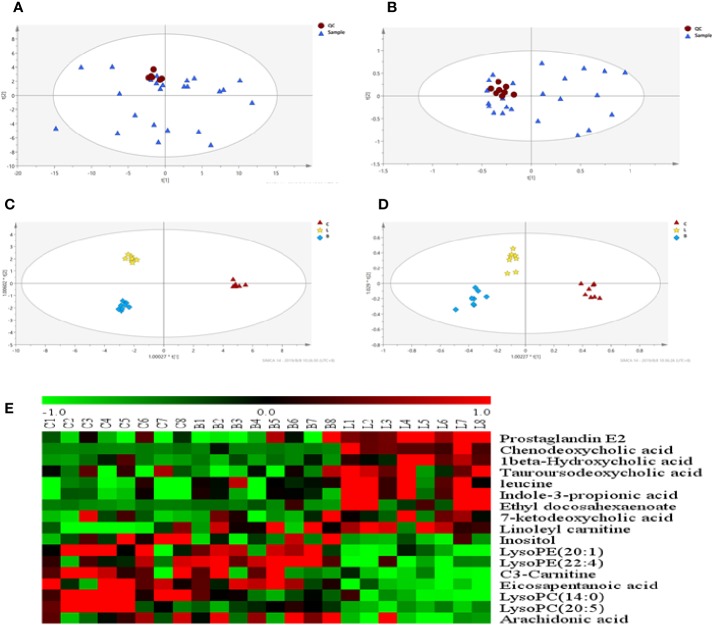
Effect of BZYQD intervention on serum metabolic profiling in loperamide-treated rats. **(A, B)** PCA score plot in three groups, R^2^X = 0.513, Q^2^ = 0.41 in positive model. R^2^X = 0.567, Q^2^ = 0.279 in negative model. **(C, D)** OPLS-DA score plot in three groups, R^2^X = 0.788, R^2^Y = 0.995, Q^2^ = 0.515 in positive model. R^2^X = 0.544, R^2^Y = 0.918, Q^2^ = 0.487 in negative model. **(E)** Hierarchical clustering heat map of the differential metabolite levels in control (C), loperamide (L), and loperamide + BZYQD groups (B) (n = 8). The heat map indicated that the serum levels of nine metabolites (Green color) were decreased, and eight metabolites (Red color) were increased in loperamide + BZYQD group as compared with loperamide group.

**Figure 5:**
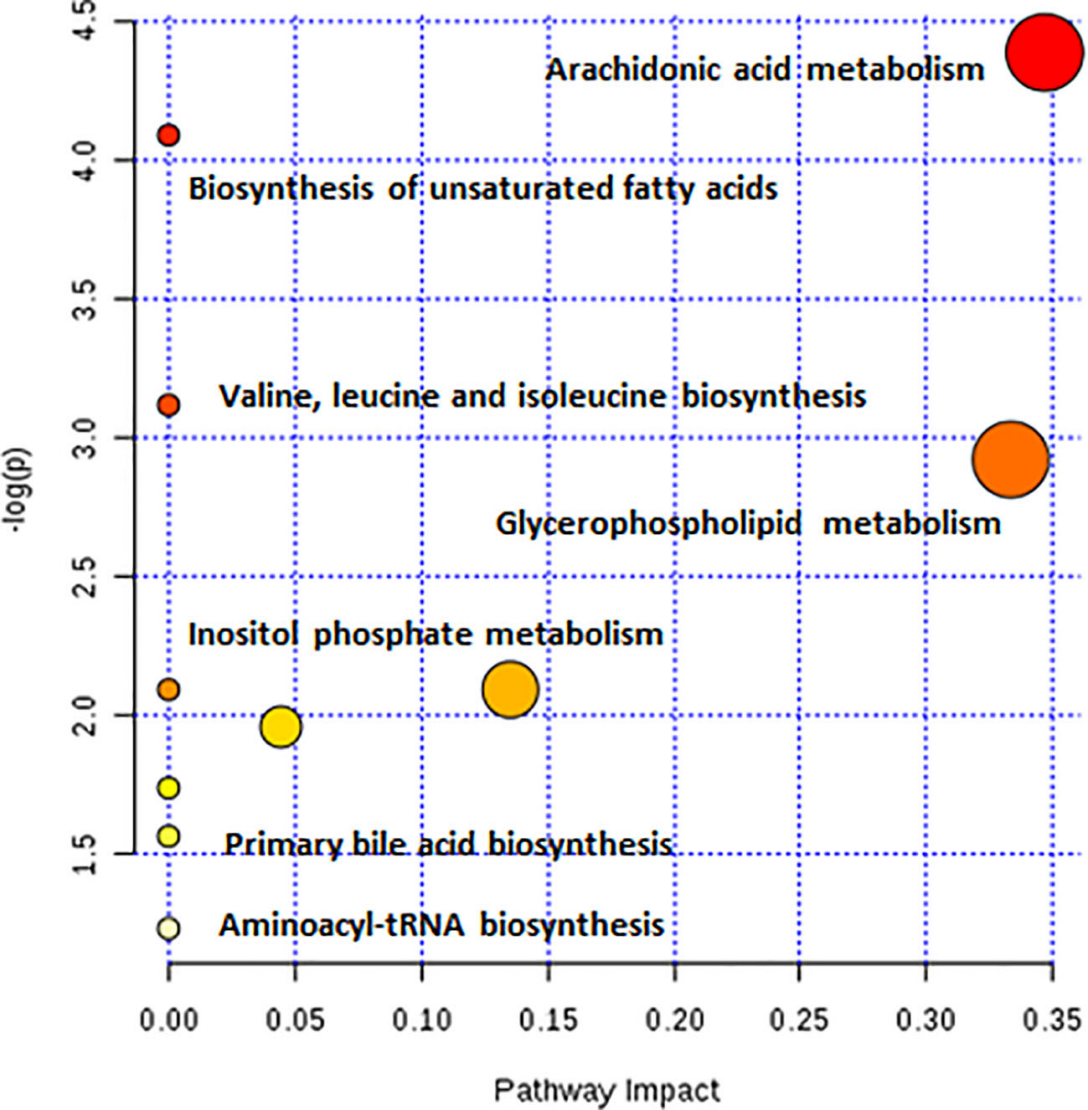
Main pathway analysis associated with metabolites that were influenced by BZYQD.

As observed in the PCA plots, the QC samples were clustered closely under both positive and negative monitoring modes, which suggested a stable analysis method (**Figures 4A, B**). The OPLS-DA results indicated an appreciable separation of the data relating to these three groups (**Figures 4C, D**). After identification and screening, levels of 29 serum metabolites in the loperamide induced group were significantly different from those in the normal control group, and BZYQD treatment reversed levels of 17 of these metabolites (**Table 3**). Among these differential metabolites, four were identified using authenticated standards and others were deduced using accurate molecular weights and comparing tandem mass spectrometry fragments with data in metabolomics databases. The levels of metabolites in the three groups are presented as a heatmap (**Figure 4E**). To further explore the metabolic pathways that BZYQD influenced, these metabolites were imported into MetaboAnalyst (https://www.metaboanalyst.ca/) to conduct pathway analysis. The related metabolic pathways are listed in **Figure 5** and **Table 4**. The metabolic pathways influenced by BZYQD were arachidonic acid (AA) metabolism, biosynthesis of unsaturated fatty acids, ascorbate and aldarate metabolism, alactose metabolism, and inositol phosphate metabolism. According to the p value and –log (p) (Zhang et al., 2015; Wu et al., 2017), arachidonic acid metabolism is one of the main pathways that influenced by BZYQD.

**Table 3:** Endogenous metabolites identified in the serum of rats included in this study.

VIP	Time	Molecular ion	CompMW	Formula	Metabolites
1.0830	8.22	[M-H]^-^	352.47	C_20_H_32_O_5_	Prostaglandin E2
1.6241	8.16	[M-H]^-^	304.47	C_20_H_32_O_2_	Arachidonic acid
2.1715	6.29	[M-H]^-^	392.57	C_24_H_40_ O_4_	Chenodeoxycholic acid
1.2442	6.37	[M-H]^-^	424.28	C_24_H_40_O_6_	1beta-Hydroxycholic acid
1.8625	5.91	[M-H]^-^	499.70	C_26_H_45_NO_6_S	Tauroursodeoxycholic acid
1.2511	8.33	[M-H]^-^	180.16	C_6_H_12_O_6_	Inositol
1.4063	8.89	[M-H]^-^	549.37	C_28_H_56_NO_7_P	LysoPC(20:1)
1.1752	7.97	[M+H] ^+^	571.17	C_30_H_52_ NO_7_P	LysoPC(22:4)
1.9844	5.31	[M+H]^+^	131.17	C_6_H_13_NO_2_	Leucine
2.0029	5.31	[M+H]^+^	189.21	C_11_H_11_NO_2_	IpA(Indole-3-propionic acid)
1.5352	1.11	[M+H]^+^	161.20	C_7_H_15_NO_3_	Carnitine
1.2700	8.70	[M+H]^+^	302.22	C_20_H_30_O_2_	Eicosapentanoic acid
2.6147	6.39	[M+H]^+^	356.27	C_24_H_36_O_2_	Ethyl docosahexaenoate
1.5642	6.14	[M+H]^+^	406.27	C_24_H_38_O_5_	7-ketodeoxycholic acid
1.7325	8.11	[M+H]^+^	423.33	C_25_H_45_NO_4_	Linoelaidyl carnitine
1.3189	7.58	[M+H]^+^	467.29	C_22_H_46_NO_7_P	LysoPC(14:0)
1.7046	7.63	[M+H]^+^	541.32	C_28_H_48_NO_7_P	LysoPC(20:5)

**Table 4:** The metabolic pathways that influenced by BZYQD in loperamide-treated rats.

Pathway Name	Total	Hits	p	-log(p)	Impact
Arachidonic acid metabolism	36	2	0.012167	4.409	0.3468
Biosynthesis of unsaturated fatty acids	42	2	0.016393	4.1109	0.0000
Ascorbate and aldarate metabolism	9	1	0.043713	3.1301	0.0000
Galactose metabolism	26	1	0.12183	2.1052	0.0000
Inositol phosphate metabolism	28	1	0.13065	2.0353	0.1116
Glycerophospholipid metabolism	30	1	0.13939	1.9705	0.0444
Primary bile acid biosynthesis	46	1	0.20667	1.5767	0.0298

### Effect of BZYQD on Inflammatory Response in Rats with Loperamide-Induced Constipation

**Figure 6:**
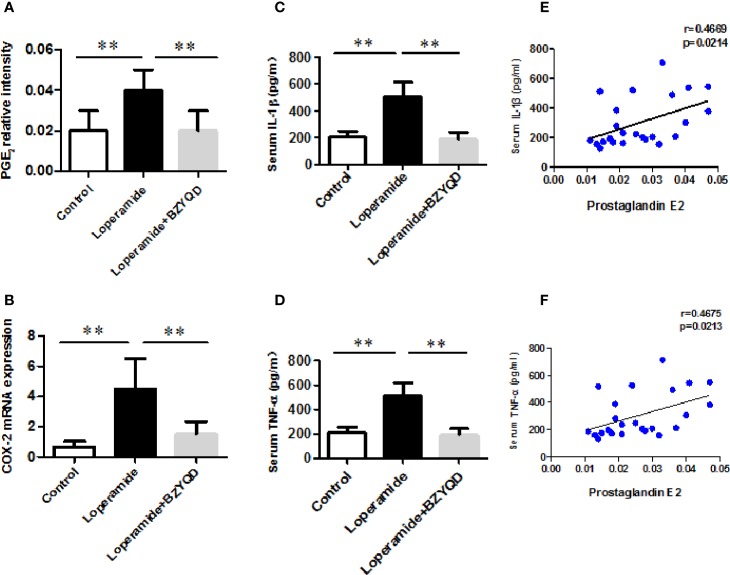
Effect of BZYQD intervention on serum levels of inflammatory factors in loperamide-treated rats. **(A)** Serum levels of prostaglandin E_2_ (PGE_2_) in each group; **(B)** The mRNA expression of COX-2 in mice colon tissue; **(C)** Serum levels of IL-6 detected using ELISA in each group; **(D)** Serum levels of TNF-α detected using ELISA in each group; **(E)** The correlation between serum levels of IL-6 and serum metabolite PGE_2_; **(F)** The correlation between serum levels of TNF-α and serum metabolite PGE_2_; data are represented as mean ± SD (n = 8). **P < 0.01 between two groups. Correlation factor (r) and *P*-value were determined using the Pearson's correlation analysis.

**Figure 7:**
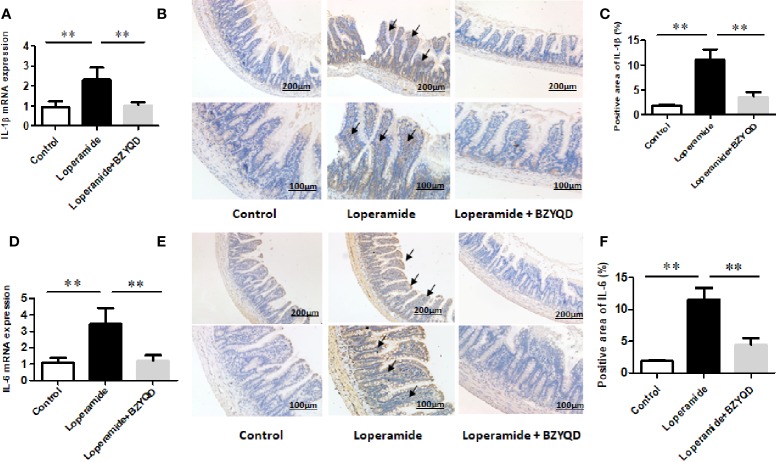
Effect of BZYQD intervention on expression of colon inflammatory factors in loperamide-treated rats. BZYQD decreased the mRNA and protein expression of IL-1β and IL-6: **(A)** The mRNA expression of IL-1β of colon tissues that detected by real-time PCR; **(B)** Protein expression of IL-1β of colon tissues that detected using immunohistochemistry; **(C)** Quantification of protein expression of IL-1β of colon tissues. **(D)** The mRNA expression of IL-6 of colon tissues that detected by real-time PCR; **(E)** Protein expression of IL-6 of colon tissues that detected using immunohistochemistry; **(F)** Quantification of protein expression of 6 of colon tissues. Data are represented as mean ± SD. Arrow marks pointed to positive area expression. (n = 5). **P < 0.01 between groups.

As serum metabolomics revealed that the main pathway influenced by BZYQD was AA metabolism, we further investigated the effect of BZYQD on inflammation in loperamide-induced rat colon tissue. As shown in **Figures 6A, B**, level of serum metabolite PGE_2_ was significantly increased in Loperamide group compared to that in the Control group, and it decreased by BZYQD treatment. Additionally, expression of cyclooxygenase 2 (COX-2), the key enzyme that produces PGE_2_ from AA was also increased in Loperamide group and decreased in BZYQD group. Moreover, the serum levels of inflammatory markers, such as IL-1β and TNF-α, were significantly increased in Loperamide group as compared with Control group. However, treatment with BZYQD decreased the serum levels of IL-1β and TNF-α (**Figures 6C, D**). Additionally, since differential metabolites, such as PGE_2_, play an important role in the progression of inflammation, correlation between serum levels of PGE_2_and those of IL-1β and TNF-α were tested using the Pearson's correlation. According to the correlation factor (r=0. 4669 or r=0. 4675) and *P*-value (p=0.0214 or p=0.0213), the results indicated serum levels of IL-1β and TNF-α had positive linear correlations with PGE_2_ levels (**Figures 6E, F**). The Pearson's correlation method was descripted as previously reported (Yan et al., 2017; Zheng et al., 2019). Additionally, the mRNA and protein expression of IL-1β and IL-6 in rat colon tissue was also investigated by real-time PCR and immunohistochemistry. As shown in **Figure 7**, the mRNA and protein expression of IL-1β and IL-6 was significantly increased in Loperamide group, and it decreased by BZYQD treatment (**Figure 7**). These results suggest that BZYQD treatment decreases loperamide-induced inflammation.

### Effect of BZYQD on NF-κB Signaling Pathway in Rats with Loperamide-Induced Constipation

**Figure 8:**
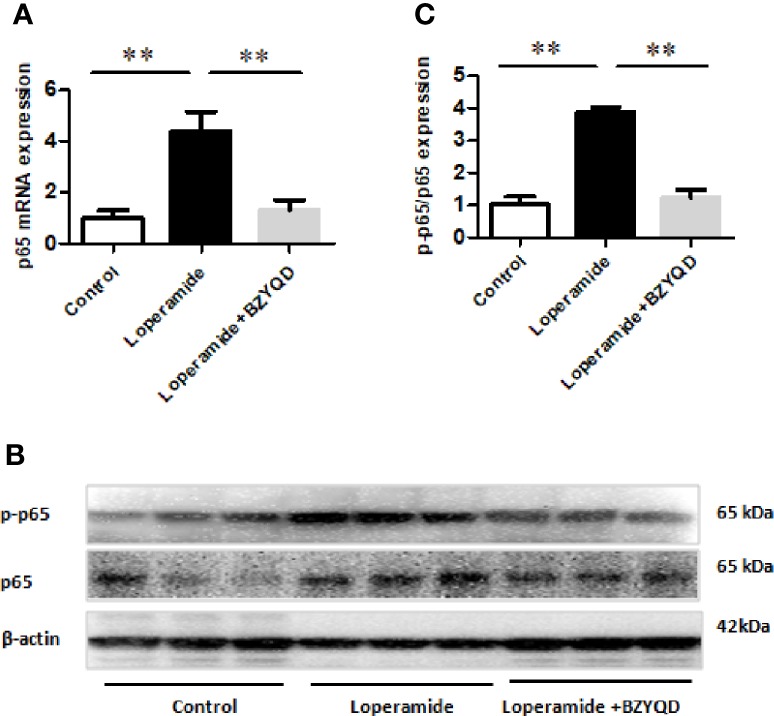
Effect of BZYQD intervention on NF-κB signaling pathway in loperamide-treated rats. **(A)** The mRNA expression of p65 detected through real-time PCR. **(B)** The protein expression of p-p65 and p65 detected by western blot. **(C)** Ratio of protein expression of p-p65 and p65; data are represented as mean ± SD; n = 8 for real-time PCR and n = 3 for western blot. **P < 0.01 between groups.

PGE_2_ is produced by AA through COX-2, which is regulated by NF-κB signaling pathway. Therefore, the mRNA and protein expression of p65 in rat colon tissue was further investigated by real-time PCR and western blotting. p65 mRNA expression and p65 and p-p65 protein levels were markedly increased in Loperamide group (**Figure 8**) than those in the control group. However, treatment with BZYQD significantly decreased p65 mRNA expression and p-p65 protein levels (**Figure 8**). This result indicated that BZYQD can regulate NF-κB signaling pathway.

### Effect of BZYQD on Serum levels of Gastrointestinal Hormone in Rats with Loperamide-Induced Constipation

**Figure 9:**
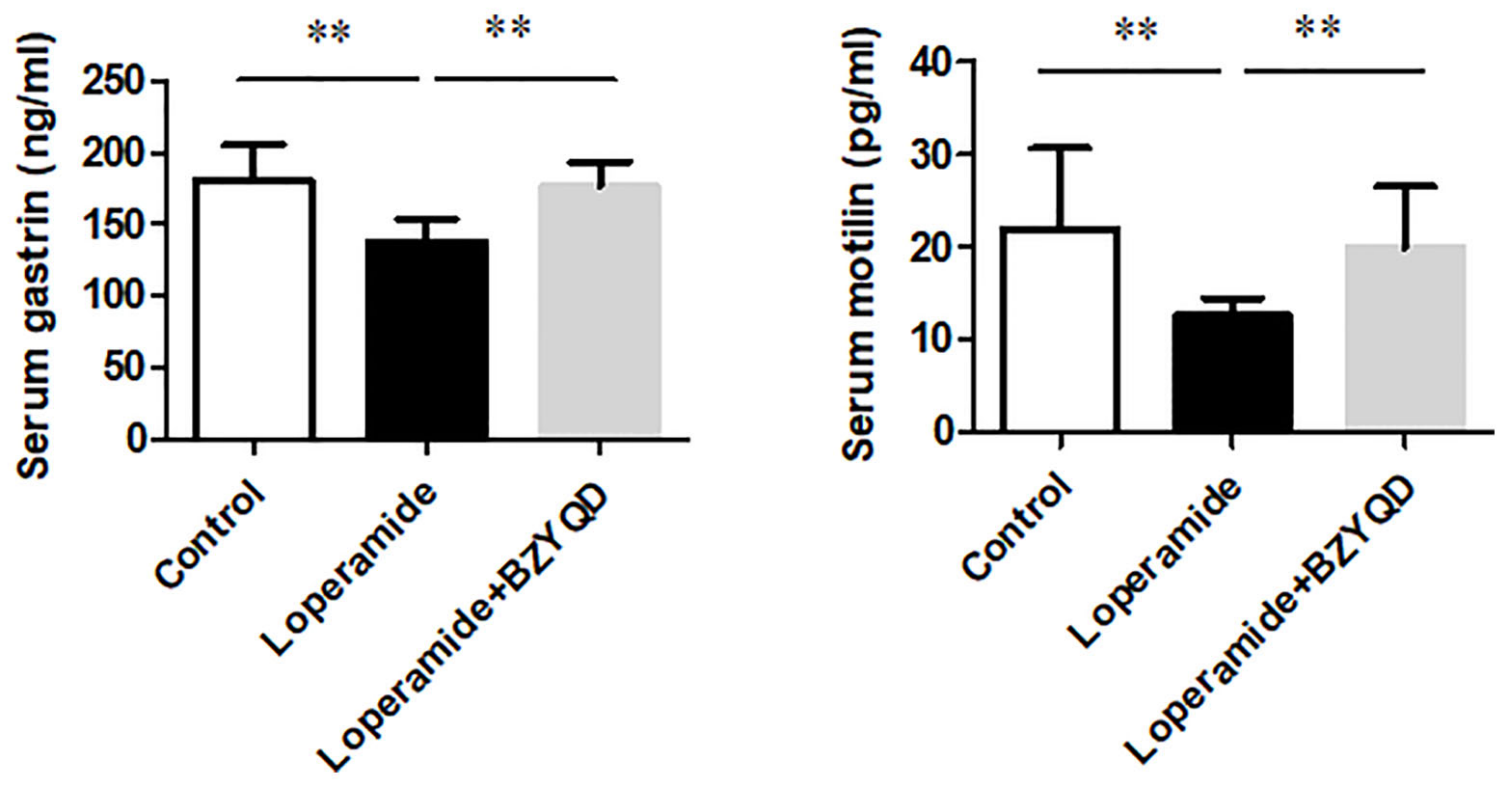
Effect of BZYQD intervention on serum levels of motilin and gastrin in loperamide-treated rats. Data are represented as mean ± SD; n = 5. **P < 0.01 between groups.

The serum levels of gastrointestinal hormone of motilin and gastrin were detected by ELISA. The serum levels of motilin and gastrin were markedly decreased in Loperamide group (**Figure 9**) than those in the control group. However, treatment with BZYQD significantly increased serum levels of motilin and gastrin (**Figure 9**). This result indicated that BZYQD can promote gastrointestinal motility.

## Discussion

BZYQD is a classic Chinese herbal formula used to treat gastrointestinal diseases (Gou et al., 2016), but its mechanism of action has not been fully studied. Loperamide is an anti-diarrheal drug commonly used in the clinic, and its mechanism of action mainly involves the inhibition of intestinal peristalsis and intestinal secretion (Zhang et al., 2018). Loperamide is widely used to establish animal constipation models to study the etiology and pathogenesis of constipation (Wu et al., 2010; Han et al., 2017; Zhang et al., 2018; Li et al., 2019). In this study, BZYQD treatment increased the intestinal propulsion rate and improved the colon tissue pathological damage induced by loperamide in a loperamide-induced rat constipation model. Mosapride is a commonly used drug to treat gastrointestinal dysfunction. Mosapride was reported to improve loperamide-induced constipation in rats (Li et al., 2019). In the current study, mosapride was used as a positive drug, and there was no significant difference between BZYQD and mosapride in improving constipation. Overall, our results indicate that BZYQD has protective effects against loperamide-induced constipation.

Metabolomics reveals molecular mechanism pathway by assessing differences in the production of metabolites or endogenous small molecules (Schrimpe-Rutledge et al., 2016). Metabolomics, thus, provides a holistic view of changes in metabolite levels due to drug interventions. TCM comprise a variety of crude herbs that exert a comprehensive therapeutic effect (Wu et al., 2017; Zheng et al., 2019). Metabolomics is very helpful in exploring the mechanisms of action of TCM (Wu et al., 2017; Zheng et al., 2019). In this study, metabolomics was used to analyze the influence of BZYQD on serum metabolites in a loperamide-induced constipation rat model. Our results showed that the levels of 29 metabolites were changed in the loperamide group compared with the control group, and 17 different metabolites were reversed by BZYQD. KEGG pathway analysis revealed that the major pathway that was influenced by BZYQD was the arachidonic acid metabolism, biosynthesis of unsaturated fatty acids, ascorbate and aldarate metabolism, inositol phosphate metabolism. Therefore, the regulation of those metabolic pathways may be key roles in BZYQD against loperamide-induced constipation.

Arachidonic acid metabolism plays an important role in the inflammatory network (Meirer et al., 2014). AA and prostaglandin E_2_ (PGE_2_) are both important metabolites involved in the AA metabolic pathway. AA is a type n-6 polyunsaturated fatty acid highly represented in the composition of phospholipids; moreover, it is the precursor of eicosanoids, such as PGE_2_, thromboxanes, and leukotrienes, which are long-recognized mediators of inflammation (Akasaka and Ruan, 2016). There have been reports indicating that the increased release of PGE_2_ can trigger an inflammatory response (Kawahara et al., 2015; Koeberle and Werz, 2015; Tsuge et al., 2019). In the present study, the serum metabolite PGE_2_ was significantly increased in the loperamide-induced group and decreased upon BZYQD intervention. This result suggests that BZYQD administration may alleviate the inflammatory response induced by loperamide in constipation rats. Histopathological results showed that intervention with BZYQD reduced inflammatory cell infiltration and morphological damage in the colons of rats administered loperamide. Further study showed that BZYQD intervention reduced the levels of serum inflammatory factors IL-1β and TNF-α and colonic inflammatory factors IL-1β and IL-6 in constipation rats. Therefore, the above mentioned results also support the results of our metabolomics study. Previous studies have shown that the inflammatory response is closely related to gastrointestinal motility disorders (Ali et al., 2013). Infiltration of inflammatory cells into the digestive tract is a major histological change involved in gastrointestinal motility disorders (Ali et al., 2013). In addition, the release of inflammatory mediators also leads to changes in gastrointestinal motility (Ali et al., 2013). A recent study reported that loperamide-induced rats exhibit a significant colonic inflammatory response, and anti-inflammatory agents help to exert a laxative effect on constipation in these rats (Kim et al., 2019). Thus, consistent with our result, this study showed that inflammation may play an important role in the progression of constipation. Our results also suggested that BZYQD can alleviate the inflammatory response induced by loperamide and ameliorate constipation.

AA is catalyzed by COX-2 to produce the metabolite PGE_2_ (Tsuge et al., 2019), while COX-2 expression is regulated by the NF-κB signaling pathway (Zha et al., 2014; Desai et al., 2018). In this study, we found that BZYQD can inhibit NF-κB phosphorylation in the colon of constipation rats, and down-regulate the expression of COX-2, thereby reducing the level of PGE_2_. Therefore, our results suggest that the regulation of the arachidonic acid pathway by BZYQD may occur through the inhibition of the NF-κB signaling pathway. However, further exploration through future studies is needed. In addition, the release of inflammatory mediators is also known to induce acute inflammatory cell infiltration and promote NF-κB activity (Kawai and Akira, 2007; Gambhir et al., 2015; Merga et al., 2016). Here, we observed that BZYQD inhibited the activation of the NF-κB pathway, which may also contribute to its protective effect against loperamide-induced constipation.

There have been reported that inositol phosphate is associated with gastrointestinal hormone motilin and gastrin metabolism (Fang et al., 2010; Goze et al., 2010). In the current study, we have investigated the effect of BZYQD on the serum levels of gastrointestinal hormone. Our results indicated that BZYQD increased the serum levels of gastrointestinal hormone motilin and gastrin. Therefore, those results suggested that treatment with BZYQD may promote the gastrointestinal motility of rats with loperamide-induced constipation.

In conclusion, the results of this study demonstrated that BZTQD intervention exerts a protective effect against loperamide-induced constipation, which may be associated with its role in regulation of multiple metabolic pathways.

## Data Availability Statement

All datasets generated for this study are included in the article/**Supplementary Material**.

## Ethics Statement

This study was carried out in accordance with the principles of China Regulations on the Administration of Laboratory Animals, the Decree No. 2 of National Science and Technology Commission of the People's Republic of China. The protocol was approved by the animal ethics committee of the Shanghai University of Traditional Chinese Medicine, China.

## Author Contributions

W-JJ and Z-KZ performed the experiment. W-JJ completed this manuscript. G-LD designed the experiment. X-PW guided the experiment. S-LC, D-DZ, and W-NY assisted the experiments. X-PW and G-LD revised the article.

## Funding

The authors are grateful for the financial support from the National Natural Science Foundation of China (81573862).

## Conflict of Interest

Author DZ was employed by company Gen Chim Testing Co., Ltd.

The remaining authors declare that the research was conducted in the absence of any commercial or financial relationships that could be construed as a potential conflict of interest.
