# Supplementary material for: Buzhongyiqi Decoction Protects Against Loperamide-Induced Constipation by Regulating the Arachidonic Acid Pathway in Rats
Source: Front Pharmacol. 2020 Apr 3;11:423. doi: 10.3389/fphar.2020.00423 (PMC7146620; doi:10.3389/fphar.2020.00423)

Buzhongyiqi Decoction Protects Against Loperamide-Induced Constipation by Regulating the Arachidonic Acid Pathway in Rats

Wan-Jun Ju^1, 2#^, Ze-kuo Zhao^1^, Shao-Li Chen^1^, Dan-dan Zhou^3,^, Wen-Ning Yang^4,^, Xiao-Ping Wen ^a1^*, Guang-Li Du^1^*

^1^Department of Formulaology, School of Basic Medical Sciences, Shanghai University of Traditional Chinese Medicine, Shanghai 201203, China

^2^Department of Endocrinology, Shanghai Pudong New Area Hospital of Traditional Chinese Medicine, Shanghai 201203, China

^3^GenChim Testing Co., Ltd, Shanghai 200131, China

^4^School of Chinese Materia Medica, Beijing University of Chinese Medicine, Beijing 102488, China

*** Corresponding author:**Xiao-Ping Wen, Guang-Li Du

Department of Formulaology, School of Basic Medical Sciences, Shanghai University of Traditional Chinese Medicine, 1200 Cailun Road, Shanghai 201203, China

Phone: 86-21-51322187

Email: duguangli2002@126.com (G.-L. Du);wxpglj@yahoo.com.cn (X.-P. Wen)

**Supplementary figure legends**

**Supplementary Figure 1. The body weight in each group**

Data are represented as mean ± SD; n = 8

**Supplementary Figure2.** OPLS-DA scores plot between each two groups

C: control group; L: loperamide group; B: loperamide+BZYQD group

**Supplementary Figure 1**


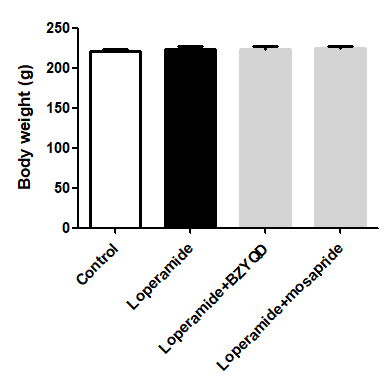


**Supplementary Figure 2**


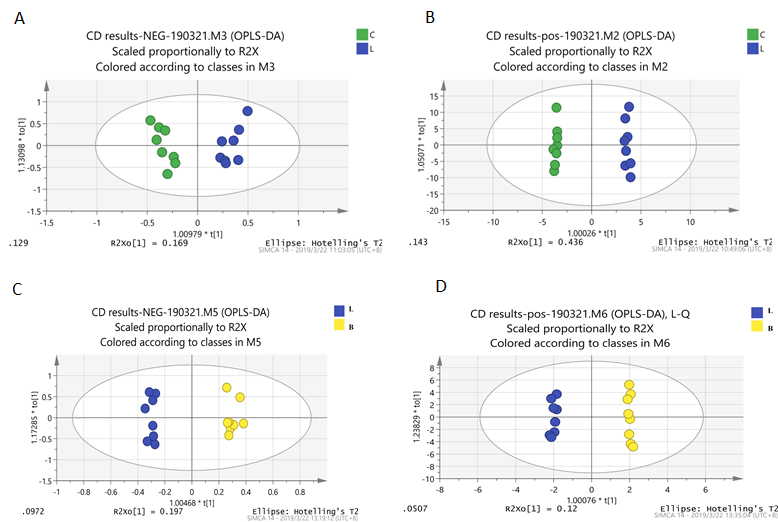

Supplement: Supplementary file 1 [file DataSheet_1.docx]
